# 

**Published:** 1891-09

**Authors:** 


CONTENTS:

PAGE

ORIGINAL ARTICLES:

Recent Experiments with Mallein—A Lymph made from Cultures of the Bacillus of Glanders. By Leonard Pearson, B.S., V.M.D. 411
The Untrusworthiness of the Reports of the Government in Relation to Investigations of Animal Diseases. By Frank S. Billings, M.D. .......... 415
Recent Foreign Investigations of Swine Diseases. By Leonard Pearson, B.S., V.M.D. .......... 419
Shoulder Lump. By Williamson Bryden, V.S. .......... 423
Porrigo ("Texas Mange") in Horses. By Gerald E. Griffin, D.V.S. 426
Extra Uterine Gestation—A Criticism. By J. Bland Sutton, London. 429
Tetanus. By Dr. Claude D. Morris .......... 439
Age of the Horse, Ox, Dog, and Other Domesticated Animals. By R. S. Huidekoper, M. D., Vet. .......... 443

EDITORIAL:

Pennsylvania State Veterinary Medical Association .......... 459
The United States Veterinary Medical Association .......... 459
Neglected Opportunities—Agricultural Shows .......... 461

SELECTIONS FROM FOREIGN JOURNALS:

A Dipping Vat for Sheep .......... 462
German Reviews .......... 464

SOCIETY PROCEEDINGS:

Joint Session of Ohio and Michigan State Veterinary Medical Association .......... 468
New York State Veterinary Medical Society .......... 470
Western Iowa Veterinary Medical Associa[illegible] .......... 474
Western Pennsylvania Veterinary Medical [illegible]iation .......... 474
New Jersey State Veterinary Society .......... 475

REVIEW DEPARTMENT:

Books and Pamphlets Received .......... 476

